# Prognostic variables predict clinical outcome after decompressive craniectomy: A single institute experience; A retrospective study

**DOI:** 10.1097/MD.0000000000036876

**Published:** 2024-01-05

**Authors:** Ebtesam Abdulla, Krishna Das, Kannan Sridharan, Mohammed Waheed, Fatima Abdulla, Joseph Ravindra, Harleen Luther, Andrew Awuah Wireko

**Affiliations:** a Department of Neurosurgery, Salmaniya Medical Complex, Manama, Bahrain; b Department of Pharmacology and Therapeutics, Arabian Gulf University, Manama, Bahrain; c Department of Neurology, Salmaniya Medical Complex, Manama, Bahrain; d Medical Institute, Sumy State University, Sumy, Ukraine.

**Keywords:** decompressive hemicraniectomy, intracranial bleeding, intracranial pressure, middle cerebral artery infarction

## Abstract

Decompressive craniectomy (DC) is a well-established neurosurgical intervention in patients with high intracranial pressure who fail to respond to medical treatment. Data on predictive factors for functional outcomes in patients with DC who have malignant middle cerebral artery (MCA) infarction as opposed to intracranial hemorrhage (ICH) are scarce. Eighty-four patients who underwent DC treatment for ICH and malignant MCA infarction were examined. All patients underwent surgery in the Bahrain Salmaniya Medical Complex Neurosurgery Unit between January 2017 and June 2021. To determine whether any of these demonstrated a link to the functional outcome, radiographic factors were compared with clinical data. The postsurgical midline shift (MLS) (ICH group) showed the strongest correlation (ρ = 0.434; *P* = .006), as in the MCA infarction group as well (ρ = 0.46; *P* = .005). Further analyses using binary logistic regression with postsurgical basal cistern status and ∆ MLS, and it was observed to be statistically significant (odds ratios: 0.067, 95% CI: 0.007, 0.67; *P* = .021). The initial Glasgow coma scale, postsurgical MLS, basal cistern status, and ∆ are Measurable variables that can be used to predict outcomes in the groups with ICH and MCA infarction.

## 1. Introduction

Decompressive craniectomy (DC) is a well-established neurosurgical procedure in clinical cases where excessive intracranial pressure (ICP) poses life-threatening risks, and medical interventions are no longer sufficient.^[1–3]^ Under these circumstances, DC is an important safeguard against mortality and serious, sustained harm. Evidence from prospective randomized trials reported a 52.8% decrease in mortality in individuals with malignant middle cerebral artery (MCA) infarctions post-DC intervention.^[4–6]^

Despite the lack of class 1 evidence, DC is frequently performed in patients with elevated ICP due to intracranial hemorrhage (ICH), a practice guided by retrospective studies that report positive outcomes.^[7–10]^ Further insight into the efficacy of decompressive surgery in ICH contexts is anticipated from the impending RESCUEicp trial.^[11]^ Nonetheless, clinical outcome data, particularly in relation to predictive factors in patients with malignant MCA infarction compared with ICH patients undergoing DC, remain markedly insufficient. Existing literature suggests that age and postoperative midline shift, as detected by CT imaging, serve as predictive elements in patients with malignant MCA infarction treated with DC.^[12–14]^ An array of data regarding the reliability of prognostic factors in ICH from existing published series, particularly those involving post-DC traumatic brain injury (TBI) patients, introduces an element of variety to this research area.^[15–17]^

## 2. Aims and objectives

This study aimed to investigate clinical and radiological indicators capable of predicting functional prognosis in patients with elevated ICP following DC. Special consideration was given to the potential variations between malignant MCA infarction and ICH caused by hypertensive bleeding, TBI, or coagulopathy, given their distinct pathophysiological mechanisms that result in increased ICP. When MCA infarction occurs, DC is performed to slow the spread of the subsequent brain injury induced by a midline shift, such as brainstem ischemia or contralateral ischemic spreading.^[18]^ In contrast, damage associated with ICH is widespread and observable prior to treatment. A series of radiological attributes discernible on conventional CT scans was established in conjunction with the size of the craniectomy, and their predictive ability was assessed. Post-DC patient data were gathered based on location and treatment duration. Assessments were made concerning the patient’s requirement for tracheostomy or artificial ventilation, the likelihood of consequent chest infection, and the underlying cause of mortality.

## 3. Methods

This retrospective study included patients who underwent DC for a malignant MCA stroke or ICH. This study was conducted at the Salmaniya Medical Complex, Ministry of Health, Manama, Bahrain, between January 2017 and June 2021. Patient data, including demographic, clinical, and radiographic information as well as craniectomy size, were collected. The clinical outcomes were evaluated using various parameters. These included the Glasgow outcome scale (GOS), radiographic characteristics, patients’ postoperative destinations, and necessity for mechanical ventilation and tracheostomy. In addition, the etiology of mortality was examined.

### 3.1. Clinical data

Patient demographic information was collected, including age, sex, comorbidities, initial diagnosis, impacted side, and DC size. The decision to perform DC, as per the guidelines of the Association of Scientific Medical Societies in Germany, was reserved for patients who failed to show significant improvement following conservative management. Early surgical decompression was applied to patients with MCA infarction, whereas DC within the ICH cohort was primarily conducted after excision of acute subdural hematoma (SDH), acute extradural hematoma (EDH), or intracerebral hemorrhage. Initial patient clinical status and emergency room findings (encompassing clinical examination, laboratory results, and CT scan) informed the decision to perform DC in patients with ICH.

In cases where intracranial hypertension was suspected, a standardized protocol for conservative management was initiated immediately after arrival at the emergency room. This included hemodynamic stabilization to avert hypotension (systolic blood pressure < 90 mm Hg) and hypoxia (PaO_2_ < 60 mm Hg), controlled hyperventilation (PaCO_2_ 28–32 mm Hg) through mechanical ventilation, head elevation by 20° to 30°, sedation (employing fentanyl), and hyperosmolar therapy with mannitol (0.25–1 g/kg). CT scans identified patients with TBI manifesting as acute SDH or EDH, exerting significant mass effects on the brain (non-evacuated mass lesion), and thus were eligible for this study.

Indications for surgical evacuation and decompression were confirmed by a senior neurosurgeon through case-by-case interdisciplinary discussion. Senior neurosurgeons and neurologists have confirmed the requirement for performing DC in patients with MCA infarction. The decision to perform DC in patients with an elevated risk of malignancy was informed by a comprehensive assessment of patient characteristics (age, medical history, laboratory results), neurological status (clinical examination, glasgow coma scale [GCS]), imaging data (CT or MRI), conservative treatment outcomes, and clinical deterioration. In line with the randomized controlled study results and recommended treatment protocols, early DC (12–48 hours) was the target.

### 3.2. Surgical procedure

Surgical intervention entailed the creation of a question-mark-shaped incision in the fronto-parieto temporal region, with termination at 1 cm anterior to the tragus. Broad fronto-occipital and temporoparietal diameters were measured to ensure sufficient decompression during craniectomy. Furthermore, the base of the temporal fossa was wholly exposed to alleviate compression of the brainstem and the temporal lobe.

The postdural incision was subjected to modifications, such as the temporalis muscle and scalp flap. The neurosurgeon responsible for performing the DC determined the specific technical nuances for each case, such as the size of the DC, approach to duraplasty, and suture material employed. Following the procedure, the bone flap was preserved at −80°C.

### 3.3. Radiological parameters

The relevant parameters investigated were the status of the basal cistern, midline shift (MLS), and lesion dimensions. Axial CT slices were used to evaluate MLS at the level of the septum pellucidum pre- and postsurgery. The craniectomy diameter was measured using postoperative sagittal CT slices. The same observer performed all radiological measurements by using a preestablished methodology. To reconstruct the physiological midline and calculate the MLS, a direct line was drawn from the frontal crest to the internal occipital protuberance at the level of the septum pellucidum. The term “LS” refers to the most substantial horizontal displacement between the midline and the displaced septum pellucidum. The hemorrhage or stroke volume was computed using the ABC/2 algorithm. Measurements were taken from the same head CT slice for the greatest length (A), the perpendicular width to A (B), and the product of the slice count and slice thickness (C). Moreover, the difference between the pre- and postoperative MLS, as well as the basal cistern status (∆ basal cistern), were assessed. The basal cistern state was categorized into 3 numeric variables: patent (1), partially effaced (2), and fully effaced (3).

### 3.4. Clinical parameters

The selected variables of interest were the initial GCS score, pupillary size and responsiveness, and the GOS at discharge. The gathered data encompassed the initial GCS score as well as preoperative and postoperative pupillary size and responsiveness. As all postoperative patients were under ventilation and sedation, obscuring significant shifts in neurological status, no postoperative GCS data were collected. The GOS was assigned on a scale of 1 to 5: a rating of 1 denoted favorable postoperative recovery, 2 indicated moderate disability, 3 suggested severe disability, 4 characterized a vegetative state, and 5 indicated mortality.

### 3.5. Statistical analysis

Demographic characteristics were presented using descriptive statistics. The chi-square test was used to ascertain the associations among categorical variables. A comparative analysis of the numerical variables was performed using the Mann–Whitney “*U*” test. Correlations between numerical variables were investigated using the Spearman correlation test, and Spearman rho (ρ) was used to compute the effect estimates. For categorical outcomes, binary logistic regression analysis was performed. Odds ratios (OR) and their corresponding 95% confidence intervals were presented as measures of effect size to illustrate the robustness of the associations. The threshold for statistical significance was set at *P* value < .05. Statistical analyses were conducted using IBM SPSS Statistics for Windows, Version 27.0, released by the IBM Corp. Armonk, NY, in 2020.

## 4. Results

In total, data from 126 participants were processed over a 54-month period. The following step involved eliminating any procedures that could not be compared because of the surgical techniques (Bifrontal DC and suboccipital DC), patient requirements (age 18 years, absence of mass lesion on preoperative CT scan, unrecorded data), type of mass effect (abscess, tumor, arachnoid cyst), or death before admission to the ICU. For this analysis, a total of 84 patients (67 men (79.76%) and 17 women (20.23%)) who presented a full set of data and were accessible for follow-up were included.

Years (range:45.4 (20–90) years. ICH (n = 28), SDH (n = 8), ICH with SDH (n = 5), ICH with EDH (n = 2), ICH with IVH (n = 1), ICH with SDH and IVH (n = 1), and SDH with EDH (n = 2) occurred in 47 patients, followed by malignant MCA infarction in 36 patients (42.85%). ICH was hypertensive in 24 patients (51.06%), TBI in 22 patients (45.83%), and coagulopathy in 1 patient (2.08%). The majority of the study participants (50, 59.52%) had right-sided DC, and 34 (40.47%) had it on the left side.

There were no statistically significant differences between the diagnosis groups regarding the following variables: sex, age, affected side, GOS, defect size, preoperative MLS, and preoperative basal cistern status. However, initial GCS, postsurgical MLS, ∆MLS, and postsurgical basal cistern were significantly different between the groups.

The predefined parameters (MLS preop and MLS postop) were tested for their prognostic significance using Spearman correlation analysis. In the ICH group, postsurgical MLS showed the strongest correlation (ρ = 0.434; *P* = .006), as in the MCA infarction group as well (ρ = 0.46; *P* = .005). Based on these findings we performed further analyses using binary logistic regression with postsurgical basal cistern status and ∆ MLS, and it was observed to be statistically significant (OR: 0.067, 95% CI: 0.007, 0.67; *P* = .021). MLS preop and preoperative basal cistern status were not further analyzed due to their inferior correlation.

The functional outcome was assessed at the end of rehabilitation, with an average of 33 (3–225) days after DC. Subsequent analyses focused on the diagnosis groups separately, each subdivided by the outcome (GOS ≤ 3 and > 3). In Table 1 The characteristics of the diagnosis groups are displayed separately according to the outcomes in Table 1. There were no statistically significant differences between these groups with respect to age, sex, affected side, defect size, initial GCS score, or pupillary reaction.

**Table 1:** Characteristics based on the outcome (GOS).

Characteristic		ICH (N = 48)		MCA-I (N = 36)		*P* value ICH	*P* value MCA-I
		GOS ≤ 3 (N = 21)	GOS > 3 (N = 27)	GOS ≤ 3 (N = 12)	GOS > 3 (N = 24)		
Sex (n [%])							
Male		20 (95.2)	21 (77.8)	7 (58.3)	19 (79.2)	.09	.7
Female		1 (4.8)	6 (22.2)	5 (41.7)	5 (20.8)		
Age (yr)		39 (20 to 80)	44.3 (20 to 80)	47.5 (20 to 70)	51.25 (20 to 90)	.6	.8
Side [n (%)]							
Right		12 (57.1)	18 (66.7)	6 (50)	14 (58.3)	.5	.09
Left		9 (42.9)	9 (33.3)	6 (50)	10 (41.7)		
GCS		10.8 (3 to 15)	9.2 (3 to 15)	14 (9 to 15)	13.2 (7 to 15)	.2	.5
Size of craniectomy (cm)		11.5 (9 to 13)	11.7 (9 to 15)	11.75 (8 to 14)	11.7 (10 to 15)	.9	.7
MLS preop (cm)		1.1 (0.3 to 1.6)	1.1 (0.5 to 1.5)	1 (0.5 to 1.8)	1.15 (0.4 to 1.7)	.87	.7
MLS postop (cm)		0.4 (0 to 1)	0.4 (0 to 1.2)	0.55 (0 to 1.9)	0.6 (0 to 2)	.85	.6
∆MLS (cm)		−0.7 (−1.2 to 0)	−0.4 (−1.4 to −0.1)	−0.3 (−1.4 to 0.3	−0.45 (−1 to 0.8)	.5	.7
Basal cistern preop (n [%])	Patent	4	3	1	0	.2	.001*
	Partially effaced	0	3	6	1		
	Effaced	17	21	5	23		
Postoperative cistern (n [%])	Patent	17	11	7	7	.39	.2
	Partially effaced	3	5	2	3		
	Effaced	1	2	3	14		

Values are expressed in mean (range) unless specified otherwise.

GCS = Glasgow coma scale, GOS = Glasgow outcome scale, ICH = intracranial hemorrhage, MCA = middle cerebral artery, MLS = midline shift, SDH = subdural hematoma.

* Statistically significant.

Moreover, binary logistic regression analysis did not reveal any significant predictive power of ∆ MLS in malignant MCA infarction patients (OR: 0.8, 95% CI: 0.2, 3.7; *P* = .7), and ICH patients (OR: 2.1, 95% CI: 0.5, 8.6; *P* = .3). Similarly, postsurgical basal cistern status did not show any statistically significant differences (OR: 1.5, 95% CI: 0.4, 5.8; *P* = .5) with the outcome in the malignant MCA infarction and in the ICH group (OR: 3.8, 95% CI: 0.8, 17.9; *P* = .09).

## 5. Discussion

In situations where a patient’s ICP is intractable, rescue therapy via DC is often employed.^[2,3]^ Decompression has been demonstrated to be beneficial in extensive prospective studies of MCA infarction, although evidence for ICH, particularly those precipitated by hypertensive hemorrhage and TBI, remains unclear.^[12,14]^ This study aimed to substantiate the relevance of prognostic factors for malignant MCA infarction and ICH. Despite representing fundamentally different causes of elevated ICP that may result in DC, the current body of evidence presents a contentious perspective.

Our observations identified postoperative MLS, basal cistern status, and MLS as strong predictive radiological markers of MCA infarction and ICH. An increase in MLS, preservation of the postoperative basal cistern, and a decrease in postsurgical MLS significantly lowered the probability of favorable outcomes. However, neither group discovered substantial value in the predictive usefulness of preoperative radiological indicators. A potential explanation is that ICH, a variable and poorly defined component of diffuse brain injury, is present even in focal injuries such as SDH or EDH.^[7]^ In contrast, MCA occlusion begins as a focal injury, but advances to ischemia owing to postoperative neurointensive care regarding hydration and blood pressure control. This effect is encapsulated by postoperative radiological parameters, as they account for swelling of both the affected and unaffected hemispheres.^[18]^

In younger patients with MCA infarction, a pooled analysis of 3 randomized controlled trials indicated a definitive benefit in DC; however, the benefits were not replicated in the older population in the DESTINY II study.^[5]^ Consequently, DC are typically administered to individuals < 60 years of age. In the context of our investigation, age was not a prognostic factor influencing the clinical outcomes of individuals with MCA infarction, with a mean age of 50 years. The use of age as a prognostic determinant in patients with ICH subjected to DC remains controversial.^[1–8]^ In contrast, a meta-analysis by De Bonis et al^[19]^ (2010) found no association between younger age and improved clinical outcomes. Concurrently, Pompucci et al^[20]^ (2007) reported inferior outcomes in patients older than 65 years but found no significant difference in clinical outcomes between patients aged 40 to 65 years and those aged < 40 years. These conclusions align with our findings, considering that the mean age of the ICH group was 42.2 years, with no statistically significant difference between favorable and unfavorable outcomes.

The initial GCS score was previously suggested as a prognostic factor in patients with ICH, although findings in patients with ICH who underwent DC remain ambiguous.^[8,10]^ Our study revealed a correlation between low GCS scores at the injury scene or upon admission and poor prognosis (GOS > 3) in patients diagnosed with ICH and MCA infarction. In the context of DC timing, GCS seems to have predictive accuracy and significance in a specific patient group, where DC significantly affects the clinical trajectory. The GCS reflects the patient’s clinical status at the time of acute brain injury and serves as an initial assessment criterion. However, this may not adequately account for the further deterioration in patients undergoing DC. This observation underscores the need for additional prognostic indicators during the postsurgical phase.

Our study demonstrates that initial GCS, MLS, basal cistern status, and postoperative MLS should be considered as standard measures to evaluate the prognosis of patients with malignant MCA infarction and ICH undergoing DC. These indicators have been integrated into routine clinical practice and are critical for predicting clinical outcomes. Outcome prediction is essential in interdisciplinary intensive care settings to optimize patient satisfaction, quality of life, and family support. Notably, despite a relatively high disability level, most patients have functional status.

## 6. Limitations of study

This study has a few limitations. First, this was a single-center study, which may not accurately reflect the results of other centers across the country. Additionally, because this was a single-center study, our retrospective study included a small number of participants. Prospective multi-center studies on this topic are required to communicate more robust outcome evidence on the topic.

## 7. Conclusion

The initial GCS and postoperative radiological measurements, such as MLS, basal cistern status, and MLS, are quantifiable and reliable radiological criteria that serve as robust predictors of outcomes in patients diagnosed with malignant MCA infarction and ICH. These criteria may play a transformative role in primary management strategies and offer critical guidance to colleagues and patient families when determining therapeutic pathways. Further controlled and prospective studies are required to confirm these findings.

## Author contributions

**Conceptualization:** Ebtesam Abdulla, Krishna Das.

**Data curation:** Ebtesam Abdulla, Krishna Das, Kannan Sridharan, Mohammed Waheed, Fatima Abdulla, Joseph Ravindra.

**Formal analysis:** Ebtesam Abdulla.

**Methodology:** Ebtesam Abdulla.

**Supervision:** Harleen Luther.

**Writing – original draft:** Ebtesam Abdulla, Krishna Das, Kannan Sridharan, Mohammed Waheed, Fatima Abdulla, Joseph Ravindra, Harleen Luther, Andrew Awuah Wireko.

**Writing – review & editing:** Ebtesam Abdulla, Krishna Das, Kannan Sridharan, Mohammed Waheed, Fatima Abdulla, Harleen Luther, Andrew Awuah Wireko.
